# Association Between Cardiopulmonary Fitness and Degenerative Lumbar Spine Disease in Patients with Heart Failure: a Retrospective Study Using CPET

**DOI:** 10.7150/ijms.121138

**Published:** 2025-10-01

**Authors:** Zong-Han Lin, Sheng-Hui Tuan, Ko-Long Lin, Wen-Hwa Wang, Wan-Yun Huang, Shu-Fen Sun, Ruei-Sian Ding, I-Hsiu Liou

**Affiliations:** 1 Department of Orthopedics, Ditmanson Medical Foundation Chia-Yi Christian Hospital, Chia-Yi, Taiwan (R.O.C.).; 2 Department of Rehabilitation Medicine, Cishan Hospital, Ministry of Health and Welfare, Kaohsiung, Taiwan (R.O.C.).; 3 Department of Physical Medicine and Rehabilitation, School of Medicine, College of Medicine, Kaohsiung Medical University, Kaohsiung, Taiwan (R.O.C.).; 4 Department of Physical Medicine and Rehabilitation, Kaohsiung Medical University Hospital, Kaohsiung, Taiwan (R.O.C.).; 5 Department of Physical Medicine and Rehabilitation, Kaohsiung Municipal Siaogang Hospital, Kaohsiung, Taiwan (R.O.C.).; 6 Cardiovascular Center, Kaohsiung Veterans General Hospital, Kaohsiung, Taiwan (R.O.C.).; 7 Department of Physical Medicine and Rehabilitation, Kaohsiung Veterans General Hospital, Kaohsiung, Taiwan (R.O.C.).; 8 Institute of Allied Health Sciences, College of Medicine, National Chen Kung University, Tainan, Taiwan (R.O.C.).; 9 School of Medicine, National Yang Ming Chiao Tung University, Taipei, Taiwan (R.O.C.).; 10 Department of Physical Therapy, School of Medical and Health Science, Fooyin University, Kaohsiung, Taiwan (R.O.C.).

**Keywords:** cardiopulmonary exercise testing, degenerative lumbar spine disease, heart failure, oxygen uptake efficiency slope, six-minute walk test

## Abstract

Heart failure (HF) is a complex clinical syndrome characterized by impaired exercise capacity and reduced quality of life. While musculoskeletal conditions such as degenerative lumbar spine disease (DLSD) are common in older adults, their contribution to exercise intolerance in HF patients remains under-investigated. This retrospective cohort study evaluated the relationship between DLSD and functional capacity in HF patients using cardiopulmonary exercise testing (CPET) and the six-minute walk test (6MWT). We included 286 HF patients who underwent CPET following hospitalization for acute decompensated HF. Based on imaging findings, patients were divided into DLSD (n = 143) and non-DLSD (n = 143) groups after propensity score matching for age, sex, and BMI. The DLSD group exhibited significantly poorer exercise tolerance, with lower oxygen uptake efficiency slope (OUES) (1.05 ± 0.44 vs. 1.17 ± 0.45; p = 0.017) and shorter 6MWT distances (244.9 ± 130.36 vs. 283.36 ± 132.22 m; p = 0.014). Multivariate logistic regression adjusting for age, sex, BMI, and comorbidities revealed that higher OUES and longer 6MWT distances were independently associated with reduced odds of DLSD (OUES: OR = 0.477; 95% CI: 0.259-0.879; p = 0.018; 6MWT: OR = 0.997; 95% CI: 0.995-0.999; p = 0.01). These findings suggest that DLSD may exacerbate exercise intolerance in HF and highlight the value of CPET and 6MWT in identifying high-risk subgroups. Early recognition of DLSD may facilitate tailored rehabilitation strategies to improve clinical outcomes in patients with HF.

## 1. Introduction

Heart failure (HF) is a multifaceted clinical condition, denoting a heart abnormality that hinders sufficient blood supply to meet the body's demands. It imposes a significant global burden, affecting approximately 64 million individuals worldwide^1^, ^2^. HF is characterized by cardiac insufficiency and typical symptoms such as dyspnea, limited exercise tolerance, and reduced peripheral perfusion^3^-^5^. Exercise intolerance in HF is multifactorial, involving impaired cardiac and pulmonary reserves, diminished skeletal muscle perfusion, and reduced muscular functionality^6^. Evaluating this intolerance is crucial, given its profound impact on patients' quality of life and mortality risk, especially in the context of extended survival due to advances in HF treatment^6^.

To assess functional impairment and stratify prognosis, several tools are commonly employed in clinical practice, including the New York Heart Association (NYHA) functional classification^7^, the six-minute walk test (6MWT)^8^, ^9^, and cardiopulmonary exercise test (CPET)^10^. Among these, CPET is regarded as the gold standard for evaluating aerobic capacity and predicting event-free survival across all HF phenotypes^11^. Peak oxygen consumption (peak VO₂) is a well-established CPET parameter, yet many patients, particularly those with preserved (HFpEF) or mildly reduced ejection fraction (HFmrEF), often cannot reach maximal effort levels due to fatigue, dyspnea, or hemodynamic instability^12^. Consequently, submaximal indices such as the anaerobic threshold (AT)^13^, ventilatory efficiency (VE/VCO₂ slope)^14^, and oxygen uptake efficiency slope (OUES)^15^ have gained recognition as robust prognostic markers that are less effort-dependent and more broadly applicable.

Approximately 84% of adults have encountered lower back pain (LBP) at some stage in their lives^16^, ^17^. Among the significant contributors to LBP is degenerative lumbar spine disease (DLSD), whose prevalence increases with age^18^, potentially leading to spinal or hip pain and spinal stenosis in severe cases^19^, ^20^. Research on DLSD risk factors is limited^21^. Previous studies have suggested associations with factors, including sex, obesity, age^22^, and biomechanics^20^, ^23^. Biochemical stressors (e.g., the intestinal microbiota) may adversely impact the spinal structure cells and tissues^24^, ^25^. Emerging evidence also points to cardiovascular disease (CVD) as a potential risk factor for DLSD^26^, with mechanisms involving atherosclerosis^27^, abdominal aorta calcification^28^, ^29^, and peripheral vascular obstruction^30^. These vascular changes may reduce spinal perfusion, leading to disc degeneration and facet joint osteoarthritis (OA) via both macrovascular and microvascular ischemia^29^.

Importantly, the relationship between HF and DLSD may be bidirectional. Vascular dysfunction in HF, including reduced cardiac output and impaired peripheral perfusion, may compromise spinal blood supply and accelerate degeneration^29^. Conversely, DLSD can lead to chronic pain and reduced mobility, further contributing to physical inactivity and cardiovascular deconditioning^1^. Notably, back pain is prevalent in 67.71% of older adults with HF^31^, which has been associated with increased cardiovascular mortality^32^. DLSD often coexists with OA, another condition linked to higher risks of myocardial infarction and HF^33^. These overlapping mechanisms, such as inflammation, vascular stiffness, and inactivity, underscore the need to consider spinal health in the comprehensive care of HF patients. Despite the high prevalence of low back pain among patients with heart failure, the specific contribution of degenerative lumbar spine disease and how it alters cardiopulmonary exercise performance remains poorly defined. This study aims to bridge this knowledge gap by using CPET to objectively evaluate the exercise performance of HF patients with DLSD, correlating real-time functional capacity and spine pathology. This study seeks to improve understanding of how DLSD influences exercise capacity in HF patients and emphasize the importance of early diagnosis and intervention.

## 2. Materials and Methods

### 2.1 Study design and participants

This was a retrospective cohort study conducted at a tertiary medical center in Southern Taiwan and included patients with HF who underwent their first CPET between March 2014 and June 2023. Eligible participants were those who had been previously hospitalized at our institution for acute decompensated HF and had subsequently attended at least three outpatient follow-up visits in the departments of cardiology, cardiovascular surgery, or rehabilitation. Patients with HF with reduced ejection fraction (HFrEF), HFmrEF, and HFpEF were all included. The diagnosis of HF was based on the criteria outlined by the American Heart Association^34^, which incorporate clinical signs and symptoms of congestion (e.g., dyspnea, peripheral edema), elevated natriuretic peptide levels, and objective evidence of structural or functional cardiac abnormalities, including variations in ejection fraction. All HF diagnoses were confirmed by experienced cardiologists following a comprehensive clinical evaluation. Only data from each patient's first CPET were included in the analysis. Inclusion criteria were: (1) age ≥50 years and (2) a confirmed diagnosis of HF (3) possess a comprehensive record of a standard 12-lead electrocardiogram (ECG). Exclusion criteria included: (1) missing clinical or CPET data; (2) inability to complete CPET due to frailty or physical limitations; (3) prolonged bedridden status exceeding three months; (4) cognitive impairment or neuromuscular disorders affecting participation in rehabilitation; (5) ventilator dependence; or (6) severe pulmonary disease requiring oxygen therapy. The study was approved by the Institutional Review Board of Kaohsiung Veterans General Hospital (IRB No. VGHKS17-CT11-11). Given the retrospective nature of the study, the requirement for informed consent was waived; however, all patients had undergone CPET as part of routine clinical care and were informed of its purpose at the time of testing.

### 2.2. Cardiopulmonary exercise testing

All cardiopulmonary exercise tests (CPETs) were performed using a symptom-limited, progressive ramp protocol on a leg ergometer (MetaLyzer 3B system, Cortex Biophysik GmbH Co., Leipzig, Germany), which included an ECG monitor, gas analyzer, flow module, and leg dynamometer. The workload was incrementally increased at a rate of 10 watts per minute. Each test was supervised by a board-certified physiatrist with over 10 years of clinical experience (K.-L. Lin) and conducted by physical therapists with more than 5 years of experience in cardiac rehabilitation (W.-Y. Huang). Continuous 12-lead ECG monitoring was conducted throughout the test, along with real-time measurements of oxygen consumption (VO₂), carbon dioxide production (VCO₂), minute ventilation (VE), respiratory rate, and other derived variables such as the respiratory exchange ratio (RER), OUES, and VE/VCO₂ slope. CPET was terminated upon the appearance of intolerable symptoms such as severe dyspnea, chest pain, dizziness, extreme fatigue, or instability, or if patients reached a submaximal endpoint following the criteria set by the American College of Sports Medicine^35^. This endpoint was defined as achieving ≥75 watts workload, a peak VO₂ of ≥5 metabolic equivalents (METs), a peak heart rate ≥70% of the age-predicted maximum, or an RER ≥1.1. These modified criteria, originally adapted by Lin et al.^36^, have been effectively implemented at our institution for patients with acute myocardial infarction and HF, demonstrating their clinical applicability and safety in this population.

The AT is determined by a noticeable increase in the VCO2-VO2 slope^37^. Peak VO2 represents the highest value of oxygen uptake recorded during the test. OUES was calculated using the linear regression formula VO₂ = a·log(VE) + b, where the slope "a" represents OUES^38^. The VE/VCO₂ slope was calculated from exercise onset to a point just beyond the AT^39^. In addition, the 6MWT was administered according to the American Thoracic Society guidelines^40^. Participants were instructed to walk back and forth along a 30-meter flat corridor for 6 minutes at their own pace while attempting to cover as much distance as possible. Standardized encouragement was provided at regular intervals, and total walking distance in meters was recorded as the 6MWT result.

### 2.3 Definition of outcomes and covariates

Demographic and clinical variables, including age, height, weight, and comorbidities, were extracted from patients' medical records prior to their initial CPET. Comorbidities were identified based on International Classification of Diseases, 10th Revision (ICD-10) codes, narrative clinical documentation, medication records, and relevant imaging findings.

DLSD-related conditions, such as vertebral osteophytes, facet joint OA (FOA), and disc space narrowing due to intervertebral disc degeneration, were identified through a comprehensive review of each patient's medical chart and imaging studies. The diagnostic criteria for DLSD were based on prior systematic reviews that established strong correlations between clinical symptoms and radiographic findings^41^. Diagnosis was confirmed using plain radiography and computed tomography (CT). X-ray findings considered diagnostic included the presence of vertebral osteophytes, disc space narrowing, and FOA. CT imaging was used to further assess spinal canal stenosis and the extent of intervertebral disc degeneration^19^, ^42^. All imaging data were independently reviewed by at least two physicians, one radiologist (Dr. M.Y. Tsai, acknowledged contributor) and one rehabilitation specialist (S.F. Sun), to ensure diagnostic accuracy and consistency. Patients diagnosed with DLSD were assigned to the DLSD group (as experimental group), while those without DLSD formed the control group. To reduce baseline differences between groups, propensity score matching was performed in a 1:1 ratio based on age at index, sex, and body mass index (BMI).

### 2.4 Statistical analysis

Statistical analyses were performed using IBM SPSS Statistics for Windows, Version 27.0 (Released 2020; IBM Corp., Armonk, NY, USA). Categorical variables were compared using the chi-square test, while continuous variables were analyzed using independent t-tests to evaluate between-group demographic differences. To enhance comparability and control for potential confounding factors, propensity score matching was conducted using a nearest-neighbor matching algorithm with a caliper width of 0.2 standard deviations of the logit of the propensity score. This approach ensured a balanced comparison between the DLSD and control groups. Sensitivity analyses were performed to verify the robustness of the matching process, confirming consistency across different caliper widths.

After matching, independent t-tests were used to compare CPET-derived continuous variables between groups. Prior to applying parametric tests, the normality of continuous variables was evaluated using the Shapiro-Wilk test, and the homogeneity of variances was assessed with Levene's test. For variables showing mild deviations from normality, additional sensitivity analyses were performed using the nonparametric Mann-Whitney U test. Logistic regression analyses (both univariable and multivariable) were conducted to calculate odds ratios (ORs) and 95% confidence intervals (CIs) for variables associated with DLSD. The assumptions of logistic regression, including independence of observations, adequate sample size, absence of multicollinearity among predictors, and linearity of continuous variables in the logit, were considered and satisfied. A two-tailed P-value of <0.05 was considered statistically significant.

## 3. Results

### 3.1 Baseline characteristics of patients with and without DLSD

In this study, a total of 530 patients who underwent CPET were initially screened. After excluding individuals younger than 50 years of age (n = 70) and those with incomplete data (n = 65), 395 patients with HF were included in the final analysis. Among them, 160 patients diagnosed with DLSD were classified into the experimental group, while the remaining 235 patients without DLSD comprised the control group. Baseline demographic comparisons revealed significant differences between the two groups, most notably a higher mean age in the DLSD group (70.98 vs. 64.80 years). To improve comparability and reduce potential confounding, 1:1 propensity score matching was performed based on age, sex, and BMI, resulting in 143 matched patients per group. Post-matching statistical analysis confirmed that there were no significant differences in age, sex, BMI, or comorbidity profiles between the two groups (Table 1).

### 3.2 Comparison of cardiopulmonary parameters between DLSD and control groups

All enrolled patients were classified as NYHA functional class II or III. The exercise capacity parameters from CPET and 6MWT for patients in the DLSD and control groups are summarized in Table 2.

The DLSD group demonstrated significantly poorer cardiopulmonary performance than the control group, with lower OUES (1.05 ± 0.44 vs. 1.17 ± 0.45; p = 0.017) and shorter 6MWT distance (244.9 ± 130.36 vs. 283.36 ± 132.22 meter; p = 0.014). Although not reaching statistical significance, several other CPET indices, including anaerobic threshold VO₂ (AT VO₂), peak VO₂, and VO₂/work rate (WR) slope, showed a trend toward reduced values in the DLSD group, suggesting a trend toward generally lower cardiopulmonary performance. No significant differences were observed between groups in resting systolic or diastolic blood pressure, heart rate responses, VE/VCO₂ slope, RER, or end-tidal CO₂ tension (PETCO₂) at various time points during the test. These findings suggest that DLSD is associated with impaired exercise tolerance or may be more prevalent among patients with more advanced HF symptoms.

### 3.3 Logistic regression analysis of factors associated with DLSD

To further investigate the relationship between exercise capacity and the presence of DLSD in patients with HF, both univariate and multivariate logistic regression analyses were conducted (Table 3). In the univariate analysis, OUES and 6MWT were significantly associated with DLSD. Specifically, lower OUES was associated with increased odds of DLSD (OR = 0.527; 95% CI: 0.31-0.896; p = 0.018), and shorter 6MWT distances also correlated with higher DLSD risk (OR = 0.998; 95% CI: 0.996-1.000; p = 0.015).

To adjust for potential confounders, multivariate logistic regression analysis was performed, controlling for age, sex, BMI, and relevant comorbidities, including hypertension, diabetes mellitus, hyperlipidemia, chronic obstructive pulmonary disease (COPD), end-stage renal disease (ESRD), liver cirrhosis, and peripheral arterial occlusive disease (PAOD). After adjustment, both OUES (adjusted OR = 0.477; 95% CI: 0.259-0.879; p = 0.018) and 6MWT (adjusted OR = 0.997; 95% CI: 0.995-0.999; p = 0.008) remained independently and significantly associated with lower DLSD risk. These findings underscore the clinical relevance of submaximal and peak exercise performance measures in identifying HF patients at greater risk for coexisting spinal degenerative changes.

## 4. Discussion

In this study, we identified a significant association between DLSD and impaired cardiopulmonary fitness in patients with HF. Patients with DLSD exhibited poorer exercise capacity compared to those without DLSD, as reflected by lower OUES and shorter 6MWT distances. Furthermore, both OUES and 6MWT were independently associated with the presence of DLSD after adjusting for age, sex, BMI, and comorbidities. These findings underscore a potential bidirectional relationship between DLSD and HF, in which musculoskeletal degeneration may contribute to reduced physical performance, while impaired cardiovascular function may in turn accelerate spinal deterioration. Collectively, these results may prompt clinicians to incorporate spinal health as an integral component of cardiopulmonary assessment in patients with HF.

Exercise intolerance in patients with HF arises from multiple contributing factors, including impaired cardiac and pulmonary reserve as well as reduced skeletal muscle perfusion and function^6^. This condition has a profound impact on both quality of life and mortality^43^. CPET serves as a key prognostic tool and is widely utilized to evaluate exercise tolerance in individuals with HF^44^. Several CPET-derived parameters, such as a peak VO₂ ≤ 50% of the predicted value^45^, a ventilatory threshold <11 mL/kg/min^13^, and a VE/VCO2 slope >34^13^ have been strongly associated with reduced cardiac output, more severe HF symptoms, and elevated mortality risk. Furthermore, the OUES has emerged as a valuable predictor of outcomes, particularly in patients with end-stage HF or those unable to complete maximal exercise testing^46^, ^47^. Prior research has demonstrated a survival benefit in patients with OUES ≥ 1.6^46^, while individuals with an OUES <1.25 faced a 5.421-fold increased risk of major adverse cardiovascular events within one year^47^. In our study, the OUES was significantly lower in HF patients with coexisting DLSD compared to those without DLSD (1.05 ± 0.44 vs. 1.17 ± 0.45; p = 0.017), suggesting that this subgroup may be at heightened risk for adverse cardiovascular outcomes. Notably, that both groups exhibited mean OUES values below the 1.25 threshold, indicating an elevated risk of major adverse cardiovascular events across the entire cohort. This may be attributed to the fact that the CPET data analyzed in this study were obtained following acute decompensated HF episodes, specifically using the first post-discharge CPET, which may reflect a period of ongoing physiological recovery and functional limitation.

The 6MWT, valued for its simplicity, low cost, and high patient tolerability, is widely regarded as a practical alternative for individuals unable to undergo maximal exercise testing. In a retrospective study of patients with New York Heart Association (NYHA) class III or IV heart failure symptoms, the 6MWT was significantly associated with both mortality and hospitalization risk^48^. From a prognostic perspective, patients with chronic HF who walked less than 300 meters were found to have poorer outcomes^49^, while those walking less than 200 meters faced a markedly increased risk of death^50^. In our study, HF patients with coexisting DLSD demonstrated significantly shorter 6MWT distances compared to controls, suggesting that this subgroup may be at heightened risk for adverse cardiovascular outcomes.

DLSD, encompassing conditions such as spondylitis, osteoporotic vertebral fractures, FOA, intervertebral disk degeneration, and lumbar spinal canal stenosis due to ligamentous changes, represents a major contributor to LBP^22^, ^24^, ^51^. Emerging evidence has highlighted a strong association between degenerative joint diseases and cardiovascular pathology. For instance, an observational study involving 153 symptomatic patients with DLSD reported that 66% were diagnosed with metabolic syndrome, thereby substantially elevating their CVD risk^52^. Another population-based study found that individuals aged 50 years or older with lumbar OA exhibited a significantly higher incidence of myocardial infarction or angina compared to age-matched controls^53^. Peripheral joint degeneration appears to exert similar cardiovascular implications. A large pooled analysis of 32,278,744 individuals revealed that the prevalence of combined CVD among patients with OA was approximately 38.4% (95% CI, 37.2%-39.6%). Notably, these patients had a 2~3-fold increased risk of developing HF [Relative Risk (RR), 2.80; 95% CI, 2.25-3.49] and ischemic heart disease (RR, 1.78; 95% CI, 1.18-2.69) compared to controls^54^. These findings align with prior studies showing a consistent link between joint degeneration and cardiovascular outcomes^33^, ^55^. Moreover, beyond lower limb joints, symptomatic hand OA has also been associated with increased coronary heart disease risk, with a hazard ratio of 2.26 (95% CI, 1.22-4.18)^56^.

From another perspective, CVD has also been implicated as a contributing factor to the development of OA. Even after adjusting for various epidemiologic and cardiovascular risk factors associated with spinal degeneration, abdominal aortic calcification has been linked to a twofold increased risk of FOA^28^ and lumbar disc degeneration^28^, ^57^. Beyond aortic atherosclerosis, systematic reviews have emphasized a strong association between stenosis of the lumbar spine's feeding arteries and both disc degeneration and LBP^58^. Supporting this, studies from China have shown that individuals with a history of heart disease have a 1.4-fold increased risk of developing symptomatic knee OA (OR, 1.40; 95% CI, 1.07-1.82)^59^, ^60^.

In summary, beyond well-established biomechanical contributors, several potentially modifiable risk factors for DLSD have been identified. Evidence increasingly supports a bidirectional relationship between CVD and systemic degenerative joint disease. One proposed mechanism involves impaired nutrient delivery to the spine due to arterial stenosis, which may accelerate intervertebral disc degeneration^61^. Moreover, systemic inflammation, a known contributor to both vascular disease and OA may act as a shared pathophysiological link^62^, ^63^. Local inflammatory mediators such as cyclooxygenase-2 have been implicated in the progression of arterial calcification, atherosclerosis, and OA. In turn, OA may exacerbate systemic inflammation, potentially promoting further vascular damage^64^. These conditions also share common risk factors, including advanced age and obesity. Joint degeneration contributes to reduced mobility, physical deconditioning, and disability, which in turn elevate cardiovascular risk^65^. In severe OA, for example, patients often exhibit markedly reduced peak oxygen consumption^66^, ^67^. Functional impairment caused by arthritis-induced inactivity may ultimately lead to cardiovascular dysfunction. Our findings highlight the importance of early intervention and structured exercise programs for patients with DLSD to help preserve cardiovascular health and prevent further deterioration.

This study has several limitations. First, the homogeneity of the study population, compromising predominantly male veterans, limits the generalizability of the findings, particularly to females and non-veterans. Future studies should recruit more diverse and representative cohorts and include sex-stratified analyses to clarify potential sex-specific differences. Second, the sample size was relatively modest. Although 395 patients with heart failure were initially enrolled, the cohort decreased to 286 after propensity score matching, which may have reduced statistical power. Moreover, although the cohort included patients with HFrEF, HFmrEF, and HFpEF, subgroup analyses stratified by heart failure phenotype were not feasible. As cardiopulmonary performance and musculoskeletal interactions may vary across subtypes, larger studies are warranted to explore phenotype-specific associations. Third, it should be emphasized that, due to the retrospective observational design, the present study cannot establish a causal relationship between DLSD and impaired cardiopulmonary fitness. Nonetheless, the study identified a significant association between the two conditions. The observed associations should therefore be interpreted as correlations and prospective or interventional studies are needed to clarify causality. Finally, other musculoskeletal disorders common in patients with heart failure, such as knee or hip OA and sarcopenia, were not evaluated or excluded. These conditions may influence exercise capacity and functional performance, potentially confounding the relationship between DLSD and cardiopulmonary fitness. Future research should incorporate comprehensive musculoskeletal assessments to better delineate their impact on CPET and 6MWT outcomes.

## 5. Conclusion

This study demonstrates a significant association between DLSD and impaired cardiopulmonary fitness in patients with HF. Specifically, HF patients with DLSD exhibited lower OUES and shorter 6MWT distances compared to matched controls, suggesting poorer exercise tolerance and potentially heightened cardiovascular risk. Both OUES and 6MWT remained independently associated with DLSD after adjusting for demographic and clinical confounders. Our results support the hypothesis of a bidirectional relationship between musculoskeletal and cardiovascular health, underscoring the importance of spinal health assessment in HF management. Given the shared pathophysiological pathways, such as systemic inflammation, vascular dysfunction, and physical deconditioning, early recognition and targeted interventions for DLSD in HF patients may help preserve functional capacity and reduce long-term cardiovascular risk. Collectively, these insights may prompt clinicians to consider spinal health as an integral component of cardiopulmonary assessment in patients with HF.

## Figures and Tables

**Table 1:** Baseline characteristics of study subjects (before and after propensity score matching)

	Before matching				After matching ^a^		
	DLSD cohort (n=160)	Control cohort (n=235)	P value		DLSD cohort (n=143)	Control cohort (n=143)	P value
**Age at index(year)**							
Mean±SD	70.98±11.253	64.8±10.29	<0.01		68.99±10.04	68.73±10.07	0.833
**Sex**							
Male	173(73.6%)	115(71.9%)	0.7		106(74.1%)	104(72.7%)	0.789
Female	62(26.4%)	45(28.1%)			37(25.9%)	39(27.3%)	
**Height (cm)**	161.71±9.03	163.64±10.46	0.058		162.43±8.50	162.59±10.03	0.885
**Weight (kg)**	63.49±14.84	65.49±14.85	0.189		63.99±12.05	64.49±14.56	0.751
**BMI(kg/m^2^)**	24.07±3.57	24.16±4.05	0.826		24.08±4.08	24.12±4.04	0.955
**Comorbidities**							
Hypertension	115(71.9%)	155(66.0%)	0.214		99(69.2%)	95(66.4%)	0.61
Diabetes mellitus	84(52.5%)	101(43.0%)	0.063		77(53.8%)	67(46.9%)	0.237
Hyperlipidemia	80(50.0%)	116(49.4%)	0.901		70(49.0%)	70(49.0%)	1
COPD	10(6.3%)	15(6.4%)	0.958		9(6.3%)	14(9.8%)	0.277
ESRD	8(5.0%)	14(6.0%)	0.684		8(5.6%)	12(8.4%)	0.354
Liver cirrhosis	5(3.1%)	3(1.3%)	0.2		5(3.5%)	1(0.7%)	0.099
Pulmonary hypertension	1(0.6%)	11(4.7%)	0.021		0(0.0%)	7(4.9%)	0.007*
PAOD	9(5.6%)	9(3.8%)	0.401		8(5.6%)	8(5.6%)	1
^a^ Propensity score matching was performed on age at index, sex, body mass index							

BMI: body mass index; COPD: chronic obstructive pulmonary disease; DLSD: degenerative lumbar spine disease; ESRD: end-stage renal disease; PAOD: peripheral arterial occlusion disease * p < 0.05

**Table 2:** Comparison of cardiopulmonary exercise testing parameters between heart failure patients with and without degenerative lumbar spine disease

	DLSD cohort(n=143)	Control cohort(n=143)	P value	95%CI
SBP when rest	118.76±20.84	119.74±23.07	0.707	(-4.139 - 6.097)
DBP when rest	69.22±11.45	68.87±10.75	0.79	(-2.934 - 2.235)
HR when rest	73.69±13.38	75±13.77	0.414	(-1.846 - 4.475)
AT VO2(ml/min/kg)	8.08±2.25	8.57±2.4	0.076	(-0.0515 - 1.030)
AT Heart rate	92.92±16.29	93.59±18.2	0.745	(-3.356 - 4.685)
WATT max	45.64±24.44	48.03±22.41	0.389	(-3.066 - 7.849)
PeakVO2(ml/min/kg)	11.44±3.47	12.13±3.54	0.098	(-0.128 - 1.502)
Peak HR	106.08±21.29	107.76±23.82	0.53	(-3.580 - 6.936)
Peak VE	33.11±12.08	32.54±11.28	0.681	(-3.289 - 2.152)
Peak RER	1.09±0.09	1.08±0.1	0.319	(-0.034 - 0.011)
6MWT	244.9±130.36	283.36±132.22	0.014*	(7.894 - 69.020)
Peak SBP	145.85±28.02	145.35±28.41	0.882	(-7.065 - 6.072)
Peak DBP	72.2±13.34	73.12±14.49	0.579	(-2.326 - 4.158)
HRR	9.38±7.66	10.04±7.67	0.469	(-1.127 - 2.442)
VE/VCO2 slope	39.66±12.25	37.16±14.74	0.12	(-5.654 - 0.656)
OUES	1.05±0.44	1.17±0.45	0.017*	(0.023 - 0.231)
VO2/WR slope	6.99±2.93	7.67±3.15	0.057	(-0.022 - 1.394)
PETCO2 when rest	29.59±5.16	30.17±5.24	0.34	(-0.623 - 1.798)
PRTCO2 when AT	33.23±6.55	33.15±6.27	0.93	(-1.864 - 1.703)
PETCO2 when max	34.83±6.13	35.45±5.66	0.368	(-0.744 - 2.003)

AT: anaerobic threshold; DBP: diastolic blood pressure; DLSD: degenerative lumbar spine disease; HR: heart rate; HRR: heart rate reserve; OUES: oxygen uptake efficiency slope; PET CO₂: end-tidal carbon dioxide tension; RER: respiratory exchange ratio; SBP: systolic blood pressure; VCO₂: volume of exhaled carbon dioxide; VE: minute ventilation; VO₂: oxygen uptake; WR: work rate; 6MWT: six-minute walk test * p < 0.05

**Table 3:** Factors associated with degenerative lumbar spine disease

		Univariate			Multivariate	
	OR	95%CI	P value	OR	95%CI	P value
Age	1.003	(0.980 - 1.026)	0.832			
Sex						
Male		Ref.				
Female	0.931	(0.511 - 1.573)	0.789			
BMI	0.99	(0.945 - 1.037)	0.662			
**Comorbidities**						
Hypertension	0.88	(0.535 - 1.445)	0.613			
Diabetes mellitus	0.756	(0.475 - 1.203)	0.237			
Hyperlipidemia	1	(0.629 - 1.590)	1			
COPD	1.616	(0.676 - 3.863)	0.281			
ESRD	1.546	(0.612 - 3.903)	0.357			
Liver cirrhosis	0.194	(0.022 - 1.685)	0.137			
PAOD	1	(0.365 - 2.742)	1			
**CPET**						
OUES	0.527	(0.31 - 0.896)	0.018*	0.477	(0.259 - 0.879)	0.018*
6WMT	0.998	(0.996 - 1.000)	0.015*	0.997	(0.995 - 0.999)	0.008*

The effects of age, sex, BMI and Comorbidities (hypertension, diabetes mellitus, hyperlipidemia, COPD, ESRD, liver cirrhosis, and PAOD) were adjusted in the multivariable regression analysis COPD: chronic obstructive pulmonary disease; ESRD: end-stage renal disease; OR: odds ratio; OUES: oxygen uptake efficiency slope; PAOD: peripheral arterial occlusion disease; 6MWT: six-minute walk test * p < 0.05

## Abbreviations

HF: heart failure
DLSD: degenerative lumbar spine disease
CPET: cardiopulmonary exercise testing
6MWT: six-minute walk test
NYHA: New York Heart Association
HFpEF: heart failure with preserved ejection fraction
HFmrEF: heart failure with mildly reduced ejection fraction
AT: anaerobic threshold
VE/VCO₂ slope: ventilatory efficiency
OUES: oxygen uptake efficiency slope
LBP: low back pain
CVD: cardiovascular disease
MET: metabolic equivalent
RER: respiratory exchange ratio
VO₂: oxygen consumption
VCO₂: carbon dioxide production
VE: minute ventilation
ECG: electrocardiogram
BMI: body mass index
ICD-10: International Classification of Diseases, 10^th^ Revision
FOA: facet joint osteoarthritis
CT: computed tomography
COPD: chronic obstructive pulmonary disease
ESRD: end-stage renal disease
PAOD: peripheral arterial occlusive disease
SBP: systolic blood pressure
DBP: diastolic blood pressure
HR: heart rate
HRR: heart rate reserve
PETCO₂: end-tidal carbon dioxide tension
OR: odds ratio
CI: confidence interval
OA: osteoarthritis
RR: relative risk
